# Physiological and Nutritional Roles of PPAR across Species

**DOI:** 10.1155/2013/807156

**Published:** 2013-05-15

**Authors:** Massimo Bionaz, Gary J. Hausman, Juan J. Loor, Stéphane Mandard

**Affiliations:** ^1^Department of Animal and Rangeland Sciences, Oregon State University, 316A Withycombe Hall, Corvallis, OR 97330, USA; ^2^Animal and Dairy Science Department, University of Georgia, 246 Edgar Rhodes Center, 425 River Road, Athens, GA 30602-2771, USA; ^3^Department of Animal Sciences & Division of Nutritional Sciences, University of Illinois at Urbana-Champaign, 498 Animal Sciences Laboratory, 1207 West Gregory Drive, Urbana, IL 61801, USA; ^4^Université de Bourgogne, 7 boulevard Jeanne d'Arc, 21079 Dijon Cedex, France

There has been a tremendous amount of information produced on peroxisome proliferator-activated receptors (PPARs). The interest in PPARs was originally driven largely by their role in hypolipidemia and hepatocarcinogenesis, but it soon became evident that they played important roles in the metabolic syndrome and overall health of organisms including regeneration of tissues, differentiation, insulin signaling, overall lipid metabolism, and immune response (reviewed in [1–7]). From a nutritional standpoint, the PPARs are of extreme importance because of their ability to bind and be activated by long-chain fatty acids and their metabolites. Therefore, the PPARs are recognized as ideal candidates for therapeutic use in order to improve metabolism and overall health through diet. At present, there is substantial interest in therapeutic applications tailored to regulate PPARs via synthetic drugs (e.g., [8]), but the exploitation of dietary approaches is not a reality yet.

Most of our knowledge on PPARs has been produced by studies carried out in rodents and humans and little from other species, bovine and pig being the most studied among livestock species. The multitude of roles of PPARs and the possibility of regulating them through dietary approaches are also of interest in animal food production. Therefore, a comparative approach to bring together physiological and nutritional roles of PPARs across species appears critical.

For this reason, this special issue was dedicated to PPARs interspecies comparisons with a larger emphasis on livestock species compared to animal models or humans. Among the 6 papers published, 3 focused specifically on ruminants and one on chicken. The review from Bionaz et al. assembled all the information pertaining to ruminant PPARs, with emphasis on functions, activation, and potential targets for nutrigenomics approaches to improve animal production and wellbeing. The review underscored that the information about PPARs in ruminants accumulated quickly in the last decade owing to the recognition of their potential importance in those mammalian species. The functional comparison among ruminant, mouse, and human highlighted a similar role of PPAR isotypes on lipid metabolism between species. However, the data highlighted differences in the response to long-chain fatty acids. Monogastrics are more sensitive to unsaturated while ruminants, particularly bovine, are more sensitive to saturated long-chain fatty acids. Based on PPARs data generated in nonruminants and ruminants, they proposed an integrative and dynamic model encompassing the activation (by long-chain fatty acids) of the three PPAR isotypes in order to optimize the adaptation to lactation. Among others, they also reviewed the data supporting a role of PPAR*γ* in controlling milk fat synthesis in ruminants and demonstrated that this feature is not shared by mouse or, likely, other monogastrics. A pivotal role of PPAR*γ* in controlling milk fat synthesis was confirmed by the paper of Shi et al. published in the present special issue. Those authors demonstrated, using a combination of PPAR*γ* specific activator, gene expression, luciferase-PPRE assay, and siRNA techniques, that this nuclear receptor controls the expression of milk fat-related genes also in primary goat mammary epithelial cells.

The activation of PPAR*γ* using oral administration of 2,4-thiazolidinedione (TZD) in growing beef bulls was assessed by Arévalo-Turrubiarte et al. The authors aimed to test the effect of PPAR*γ* activation on intramuscular fat (i.e., marbling). They observed a greater amount of TZD in liver of the treated animals, demonstrating an uptake of the drug via oral administration. The TZD treatment had no effect on carcass quality but had a strong effect on the expression of all three PPAR isotypes in liver (all decreased) and in muscle (increase only of PPAR*α*). They observed also an overall increase in cell size and decrease of cellular synthesis in muscle and perirenal adipose tissue, but the opposite was observed in subcutaneous adipose. This effect was explained by higher insulin sensitivity due to the treatment. Activation of PPAR*γ* with oral administration of a TZD in growing pigs also had no significant effect on marbling, but it did increase muscle fiber oxidative capacity regardless of fiber type [9]. As for bovine, the activation of PPAR*γ* in swine may be useful to influence metabolism overall, but more studies are needed to examine this possibility. Takada and Kobayashi provided the first review of the three PPAR isotypes in poultry, particularly in chickens. Interestingly, they also provided a comparison with human PPARs both structurally and functionally. They uncovered several peculiar and unique functions in chicken PPARs and differences between chickens, and human PPARs. These data prompt for more chicken-specific studies in order to exploit the ability of PPARs to control lipid and glucose metabolism in this species.

Mandard and Patsouris reviewed recent evidence establishing that PPARs are critical regulators of inflammation in mammals. In the last decade, PPARs have emerged as modulators of inflammatory responses. Therefore, the potential therapeutic usefulness of PPAR*α* and PPAR*γ* activation in the control of obesity and diabetes-induced chronic (low-grade) inflammation has extensively been studied over the last couple of years using rodents. The authors discussed different aspects of the interaction of PPAR*γ* with adipose inflammation. In the light of the recent findings, it has become clear that, besides activating PPAR*γ* in the adipocyte, pharmacological activation of this receptor extends to a much broader range of cell types, such as T regulatory cells, which is likely beneficial in the suppression of obesity-associated inflammation in white adipose tissue, as far as rodents are concerned. The impact of the pharmacological activation of mouse PPAR*α* in the context of obesity-induced hepatic inflammation is also reviewed as well as the potential relevance of PPAR*β*/*δ* as a molecular drug target to fight liver inflammation in the case of nonalcoholic fatty liver disease. 

A growing body of evidence also indicates that PPARs are potent negative regulators of the acute-phase response in different species, ranging from mouse, rat, pig, cattle and humans. The review article by Mandard and Patsouris also expands on the potential beneficial use of PPAR (ant)agonists in the routine of livestock to prevent bacterial-induced excessive inflammatory reaction and associated diseases such as mastitis in dairy cows.

PPAR*α* is also known to be critical for energy homeostasis. In line with this, the review paper by Ringseis et al. thoroughly summarizes the implication of PPAR*α* in carnitine homeostasis in no less than six different species including rat, mouse, pig, cattle, chicken, and human. The comparative analysis performed by the authors led them to conclude that PPAR*α* displays a key regulator role in carnitine homeostasis in general. It is the process of cellular carnitine uptake, with the key role of the PPAR*α* target carnitine transporter novel organic cation transporter 2, that is particularly well conserved across the above-mentioned species.

All the papers in this special issue emphasized on one hand a similar function of PPARs among species, particularly related to lipid metabolism, but also, and more importantly, accentuated the differences and the species-specific functions and response to agonists.

In summary, the analysis of PPARs across species highlighted the following:

- interspecies conserved functional roles of those nuclear receptors (e.g., regulation of lipid metabolism and inflammation);
- the potential for therapeutic intervention through nutritional modulation of PPARs in all species in order to prevent diseases and improve animal production;
- differences between species that prompt for more species-specific studies in order to fully exploit the abovementioned therapeutic roles through nutrition.

*Massimo Bionaz* *Massimo Bionaz* *Gary J. Hausman* *Gary J. Hausman* *Juan J. Loor* *Juan J. Loor* *Stéphane Mandard* *Stéphane Mandard*
